# Paired Root-Soil Samples and Metabarcoding Reveal Taxon-Based Colonization Strategies in Arbuscular Mycorrhizal Fungi Communities in Japanese Cedar and Cypress Stands

**DOI:** 10.1007/s00248-023-02223-9

**Published:** 2023-04-28

**Authors:** Akotchiffor Kevin Geoffroy Djotan, Norihisa Matsushita, Kenji Fukuda

**Affiliations:** Graduate School of Agricultural and Life Sciences (Laboratory of Forest Botany), University of Tokyo, 1-1-1, Yayoi, Bunkyo-ku, Tokyo, 113-8657 Japan

**Keywords:** Mycorrhiza, AMF community, Fungal ecology, Intraradical extraradical, AMF strategies

## Abstract

Arbuscular mycorrhizal fungi (AMF) in the roots and soil surrounding their hosts are typically independently investigated and little is known of the relationships between the communities of the two compartments. We simultaneously collected root and surrounding soil samples from *Cryptomeria japonica* (Cj) and *Chamaecyparis obtusa* (Co) at three environmentally different sites. Based on molecular and morphological analyses, we characterized their associated AMF communities. Cj was more densely colonized than Co and that root colonization intensity was significantly correlated with soil AMF diversity. The communities comprised 15 AMF genera dominated by *Glomus* and *Paraglomus* and 1443 operational taxonomic units (OTUs) of which 1067 and 1170 were in roots and soil, respectively. AMF communities were significantly different among sites, and the root AMF communities were significantly different from those of soil at each site. The root and soil AMF communities responded differently to soil pH. At the genus level, *Glomus* and *Acaulospora* were abundant in roots while *Paraglomus* and *Redeckera* were abundant in soil. Our findings suggest that AMF colonizing roots are protected from environmental stresses in soil. However, the root-soil-abundant taxa have adapted to both environments and represent a model AMF symbiont. This evidence of strategic exploitation of the rhizosphere by AMF supports prior hypotheses and provides insights into community ecology.

**Supplementary Information:**

The online version contains supplementary material available at 10.1007/s00248-023-02223-9.

## Introduction

Arbuscular mycorrhizal fungi (AMF) are ubiquitous symbiotic microorganisms that live in both the soil and in roots of their hosts upon which they bestow diverse benefits [1, 2]. AMF are a monophyletic group of fungi in the Glomeromycota or Glomeromycotina [3, 4]. These fungi have wide host ranges and are obligate plant symbionts [5], which hampers investigation of their community ecology. The development of high-throughput sequencing tools has made studies of plant-microbe interactions possible without the need for culture [2, 6].

AMF communities and species richness may be similar or dissimilar between the roots and surrounding soil [7]. Different AMF communities in roots and surrounding soil may be a result of differences in, for example, strategic intraradical versus extraradical biomass allocation, sampling season, site conditions, host species, and biological material (spore or hyphae) [2, 8]. Paired root-soil paired samples of host plants collected from natural ecosystems and characterization of the associated AMF communities would provide insights into ecological patterns [2]. Such an approach may also shed light on fungal colonization strategies.

Among the few studies that compared AMF community composition between roots and surrounding soil, only those by Faghihinia et al. [9], Ji et al. [7], and Djotan et al. [10] were based on Illumina’s next-generation amplicon sequencing (NGS). Also, except for the woody host plants *Camellia japonica* [11], *Juglans mandshurica* [7], and *Cryptomeria japonica* (Japanese cedar) [10], most studies focused on annual or perennial herbs. Such studies were carried out at local scales and only one provided evidence that the intraradical AMF community originated from the roots of host plant species (*Cryptomeria japonica*) [10].

Many AMF exhibit host specificity and some host plants select AMF from an AMF pool in soil [12]. AMF are obligate symbionts, and intra- and extraradical AMF communities are typically distinct. However, plants preferentially supply photosynthate to AMF taxa that deliver the most phosphorus [13]. The structure and composition of the root-soil AMF communities that maintain the mutually beneficial associations between hosts and symbionts remain to be characterized.

In this study, we performed plant barcoding and NGS-based metabarcoding of fungal DNA from two related, co-planted, and important forest tree species in Japan. We hypothesized that any differences between the root and soil AMF communities of host plants are related to AMF taxon-based colonization strategies [14]. To test this hypothesis, we collected paired root and soil samples at three different sites with different environmental conditions, molecularly confirmed root identity, and morphologically analyzed root colonization. Next, we used NGS to characterize and analyze the composition and structure of the AMF communities in and between the roots and surrounding soil.

*Cryptomeria japonica* (Sugi or Japanese cedar, Cj) and *Chamaecyparis obtusa* (Hinoki or Japanese cypress, Co), which belong to Cupressaceae, were used as host tree species. They are both planted throughout Japan and their planted area is about 7 million hectares, constituting 69% of the total artificial forests in the country [15]. They occur naturally in warm to cool temperate regions of Honshu, Kyushu, and Shikoku Islands [16]. Morphotypes of arbuscular mycorrhiza (AM) have been reported in Cj and Co [17] and the AMF colonization rate of Cj root has been assessed [18]. However, no study has assessed Co root colonization. Furthermore, few studies such as those by Zou et al. [19], Matsuda et al. [20], and Djotan et al. [10] have investigated the AMF communities associated with Cj. To our knowledge, no study has compared the AMF community of the roots and surrounding soil of Cj and Co.

## Materials and Methods

### Study Sites

**Fig. 1:**
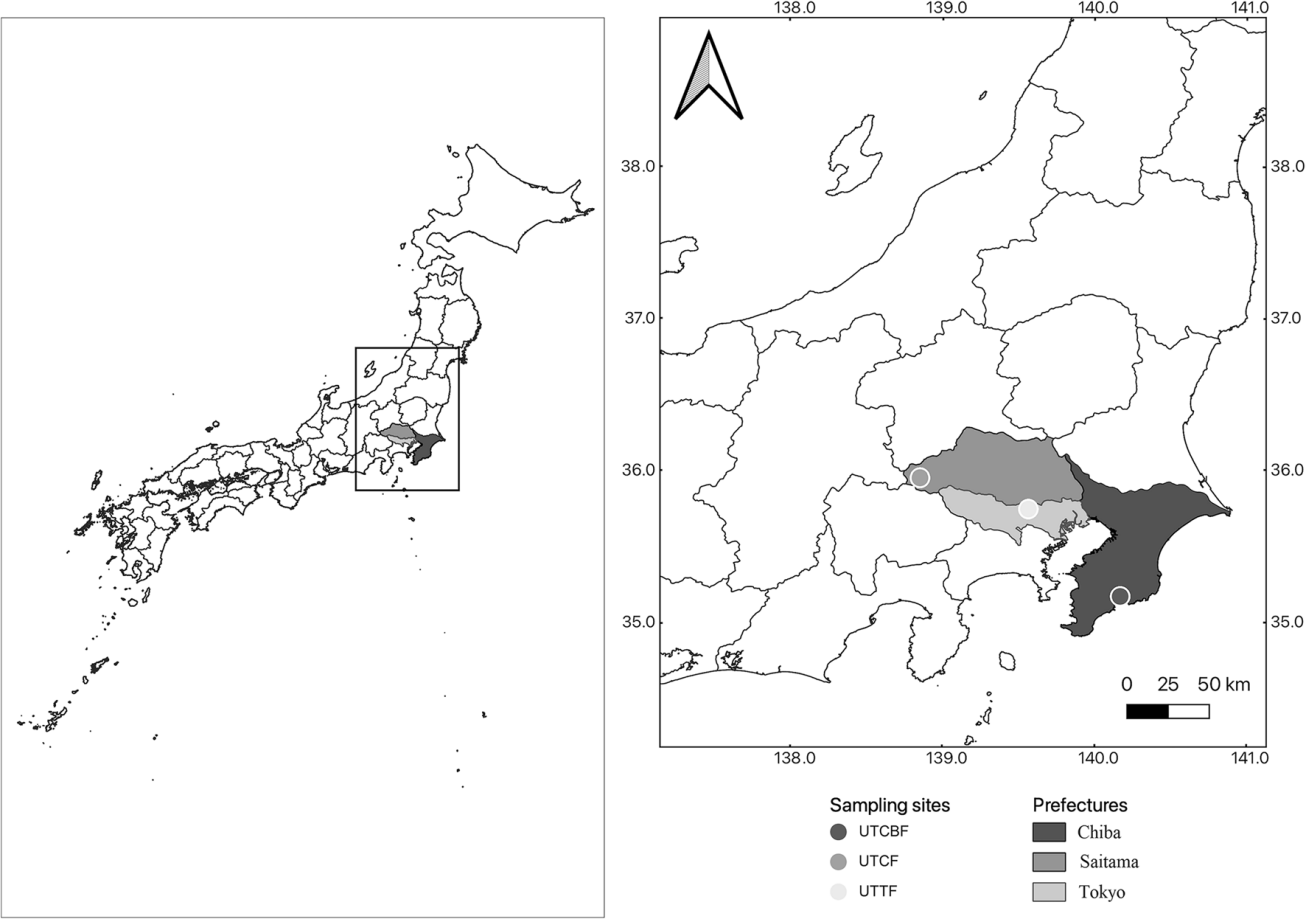
Locations of the three sampling sites. University of Tokyo Chiba Forest (UTCBF), Chichibu Forest (UTCF), and Tanashi Forest (UTTF)

We conducted this study on three Cj and Co forests in the Kanto District of Japan: The University of Tokyo Chiba Forest (UTCBF, Chiba Prefecture), Chichibu Forest (UTCF, Saitama Prefecture), and Tanashi Forest (UTTF, Tokyo Metropolitan Area) (Fig. 1). UTCBF and UTCF are located on steep slopes, whereas UTTF is on a plateau. The forests were planted between 1927 and 1983, and the stand density ranged from 600 trees/ha to 1850 trees/ha (Table S1). The diameters at breast height (DBH) of Cj and Co trees ranged from 31.5 ± 4.4 to 49.4 ± 9.0 cm (mean ± SE) and 21.8 ± 2.2 to 41.2 ± 7.1 cm, respectively (Table 1). The understories of UTCBF and UTTF plantations were covered with many shrubs and herbaceous plants. In contrast, the understory of the UTCF plantation harbored few plants because of damage by feeding of sika deer (*Cervus nippon*) (Table S2). The environment is different among sites, particularly the mean annual temperature (MAT) and the mean annual precipitation (MAP) (Table S1). Also, the forest zone, the soil, and the landscape are different between sites. UTCF is in the cool temperate region of Japan with snow depth of 20 to 30 cm. The natural forest surrounding it is composed of deciduous hardwoods. UTCBF is in the southeastern part of the Boso Peninsula and the natural forest surrounding it is composed of evergreen hardwoods. As for UTTF, it is an isolated forest patch in Nishi-Tokyo city, almost the center of the Musashino area of the Tokyo metropolitan area.

**Table 1:** Samplings and soil properties

Sites	Chiba (UTCBF)		Chichibu (UTCF)		Tanashi (UTTF)	
Host species^a)^	Cj	Co	Cj	Co	Cj	Co
Total No. of samples^b)^	10	18	16	12	20	18
DBH (cm)^c)^	49.4 ± 9.0	41.2 ± 7.1	32.6 ± 7.5	21.8 ± 2.2	31.5 ± 4.4	23.4 ± 5.6
Soil EC (μS/Cm)^c)^	130.8 ± 25.4	142.4 ± 52.9	213.5 ± 84.0	144.0 ± 54.9	174.4 ± 32.4	111.1 ±17.8
Soil pH^c)^	4.76 ± 0.35	4.46 ± 0.32	5.01 ± 0.29	4.84 ± 0.35	5.37 ± 0.12	5.05 ± 0.17

^a)^*Cj*, *Cryptomeria japonica*; *Co*, *Chamaecyparis obtusa*. ^b)^We initially collected 20 samples (10 root and 10 surrounding soil samples) per site. The number of samples corresponds to the number of samples that passed root identification and sequence processing. ^c)^Mean ± SE. DBH, diameter at breast height; EC, electrical conductivity (1 μS/cm = 1∙10^-4^ S/m). For this variable, only samples used in the community analysis were considered. Two-way ANOVA did not show a significant interaction effect between site and host species on any of the variables (Table S3)

### Sampling

We collected 60 pairs of root and soil samples in July and August 2020 (Table 1). Ten trees of each species (Cj and Co) were chosen randomly at each site. In UTCBF and UTCF, root and soil samples were collected from a mixed Cj/Co plantation, with samples collected from a Cj tree and a Co tree less than 5 m apart. In UTTF, Cj and Co were sampled from separate, adjacent pure plantations. An average of 50 g fresh roots were collected from one basal root with sufficient numbers of first- and second-order fine roots, tracked from a parent root of the target trees. Then, the surrounding soil was collected and placed with the roots into a labeled plastic bag. The samples were stored at 4°C and processed within 3 days of collection. Root and soil samples were processed as described in Djotan et al. [10] for DNA extraction, root staining, and measurement of soil pH and EC.

### Root DNA Extraction and Identity Confirmation

We extracted total genomic DNA from 15 to 18 mg milled root samples using the DNeasy Plant Mini Kit (Qiagen, Germantown, MD) according to the manufacturer’s instructions. Following Djotan et al. [10], we amplified and sequenced a 550 bp fragment of *rbc*L. The amplicon sequences were BLASTed against the NCBI GenBank database to exclude samples that did not match Cj or Co. Because paired root and surrounding soil samples were collected, the soil samples were used upon confirmation of the corresponding root samples.

### Soil Properties and DNA Extraction

We measured the pH and EC of the soil samples by adding 50 mL of sterilized distilled water to 20 g of air-dried soil that had been passed through a 1 mm sieve and shaking it for 5 min. Next, the mixtures were allowed to stand for 30 min (for pH) and 3 h (for EC). pH and EC were measured using a compact pH meter (LAQUAtwin-pH-33; Horiba, Kyoto, Japan) and a conductivity meter (LAQUAtwin-EC-33; Horiba), respectively. Total DNA was extracted from 0.1 g lyophilized soil samples added to 20 mg skim milk using the ISOIL for Beads Beating Kit (Nippon Gene, Tokyo, Japan) according to the manufacturer’s instructions.

### AMF Community Metabarcoding

DNA extracts of validated paired root/soil samples were amplified by nested PCR using KAPA2G Robust HotStart ReadyMix (KAPA Biosystems, Wilmington, DE) following Djotan et al. [10]. Briefly, primers NS1 [5′-GTA GTC ATA TGC TTG TCT C-3′] and NS4 [5′-CTT CCG TCA ATT CCT TTA AG-3′] were used in the first-round PCR, whereas primers NS31 [5′-TTG GAG GGC AAG TCT GGT GCC-3′] and AM1 [5′-GTT TCC CGT AAG GCG CCG AA-3′] whose 5′-ends were affixed to the Illumina adapters Tn5ME A [5′-TCG TCG GCA GCG TCA GAT GTG TAT AAG AGA CAG-3′] and Tn5ME B [5′-GTC TCG TGG GCT CGG AGA TGT GTA TAA GAG ACA G-3′], respectively, were used in the second-round PCR. In addition, sample-specific 6 bp index sequences were inserted between the Tn5ME A adapter and the NS31 primer to enable sample pooling before sequencing and read demultiplexing after sequencing. The final PCR products of approximately 550 bp of the small subunit ribosomal DNA (SSU rDNA) were randomly pooled by type of sample (five root or soil samples per pool) and sent to Macrogen Japan (Tokyo, Japan) for amplicon sequencing on the Illumina MiSeq platform (2 × 300 bp).

### Bioinformatics Analysis

We used QIIME2 v. 2022.2.0 [21] to process the amplicon sequences which were de novo clustered at a 97% identity threshold and the centroid sequence was selected as a representative sequence of the corresponding operational taxonomic unit (OTU). Chimera OTUs, rare OTUs (less than 10 reads across all samples), and OTUs that were detected in only one sample were discarded. The representative sequences of the remaining OTUs were annotated based on the Maarj*AM* and National Center for Biotechnology Information GenBank databases using the *NCBI-blast-2.10.0+* program. Taxa were assigned to OTUs only when from the database, both query cover and percent of identity with the match were higher or equal to 95%. Taxonomic affiliations were updated following the consensus on AMF classification [22]. The community data were normalized before all the downstream community analyses.

### Morphological Assessment of AMF Root Colonization

Five ethanol-conserved root systems were selected randomly for the assessment of mycorrhization frequency (MF) and intensity in Cj and Co. The roots were stained with Trypan blue in lactoglycerol [23]. Under a microscope, 50 randomly selected small root fragments (at least 1 cm each, 10 fragments per sample) were analyzed for each species at each site. The line interception method was used to quantify root colonization [24]. The first observation point on a given root segment was selected randomly, and at least 10 observations were performed at 1-mm intervals along that root segment, totaling at least 100 observations per sample. We calculated the MF as the proportion of the samples confirmed to contain AMF (*n* = 5 per species at a site). The mycorrhization intensities were calculated as the proportions of root sections colonized by AMF-characteristic hyphae (hyphal colonization [HC]), arbuscules (arbuscular colonization [AC]), and vesicles (vesicular colonization [VC]) following McGonigle et al. [24], except that we did not classify vesicles as hyphae but classified arbuscules as finely branched hyphae.

### Statistical Analyses

We performed statistical analysis using R v. 4.2.2 [25] software. We conducted two-way analysis of variance (ANOVA) to assess differences in soil properties (pH and EC), host DBH, and mycorrhizal colonization of roots between sites and hosts. The vegan R package v. 2.6-4 was used to estimate the alpha diversity, which we tested with ANOVA. Tukey’s honestly significant difference (HSD) test at a 95% confidence level was used to compare mean values between levels of factors that exerted significant effects on the alpha diversity. For all variables, we confirmed by Shapiro-Wilk normality test and Levene’s test for homogeneity of variance that data were normally distributed, and groups had equal variance before proceeding with parametric tests. Only EC did not conform to the requirements of parametric tests and was therefore analyzed by Kruskal-Wallis rank sum test. We calculated Pearson’s correlation using the Hmisc R package (v. 4.7-2) to assess the associations of root and soil conditions (excluding soil EC due to its non-normal distribution) with AMF root colonization.

We used a permutation-based multivariate analysis of variance (PERMANOVA) in the vegan R package to examine the effects of site, host species, and compartment on the AMF community. Similar and dissimilar communities were detected by analysis of similarity (ANOSIM) based on Bray-Curtis model in the vegan R package. The AMF community was ordinated and visualized using the ggplot2 R package v. 3.4.0. Next, we applied the multinomial species classification method (CLAM) in the vegan R package to identify the AMF OTUs and genera in each compartment of the rhizosphere (root or soil) and those significantly associated with a host (Cj or Co) [26]. We also tested the effect of soil properties (Euclidean distances in vegan for pH and EC) and geographical separation (Haversine distance in the geosphere R package v. 1.5-18) on the composition and structure of the AMF community using the Mantel test in the vegan R package.

The sequences of the top 10 most abundant OTUs (dominant) of each group of samples were aligned using MEGA11 and their maximum-likelihood phylogenetic positions were determined using an automatic model finder, tested with PhyML (SH-aLRT) and ultrafast (UFBoot) bootstraps over 1000 randomizations, all implemented in IQ-TREE 2 [27]. *Paraglomus occultum* AJ276082 served as the outgroup in the phylogenetic tree for which we relied on the clade only when its SH-aLRT ≥ 80% and UFBoot ≥ 95%. The tree was annotated and displayed using Interactive Tree of Life (iTOL, v. 5) [28].

## Results

### Soil Properties

The soil pH was significantly different between sites and host species but the interaction between the two factors was not significant (Table 1 and Table S3). It went decreasing from UTTF to UTCBF with UTCF in between. The soil EC was, however, significantly different only between host species and its higher values were recorded for Cj (Table 1 and Table S3).

### Bioinformatics Analysis

After excluding unconfirmed samples, for the remaining 94 samples, Illumina MiSeq amplicon sequencing produced 1,114,607 amplicon sequences clustered into 108,048 OTUs. After quality filtering and sequence annotation, we obtained 555,657 Glomeromycota amplicon sequences of excellent quality that clustered into 1445 AMF OTUs. After being rarefied, the normalized AMF community data comprised 226,634 (40.79% of the total) Glomeromycota amplicon sequences in 94 (100.00%) samples and clustered into 1443 AMF OTUs. We deposited the sequence read archives in the National Center for Biotechnology Information (PRJNA714473), the representative nucleotide sequences of the AMF OTUs generated (MZ479751–MZ481498) in GenBank (SUB9891895), and the partial nucleotide sequences of *rbc*L for Cj and Co (ON156682–ON156726) in BankIt (2569115).

### Composition and Structure of AMF Communities in Root and Surrounding Soil of Cj and Co

Site, host species, and compartment significantly affected the composition and structure of the AMF community at the OTU level (Table S4). In UTCBF and UTCF, only compartment significantly affected community structure and composition. However, in UTTF, both host and compartment exerted significant effects on community structure and composition (Table S5). ANOSIM showed that the AMF community was significantly different between Cj and Co only in UTTF (Table S6).

OTU richness of the root AMF community was less than that of the surrounding soil (Online Resource 1). Of the 1443 AMF OTUs, we detected 1067 and 1170 in roots and surrounding soil, respectively. Also, the average OTU richness was significantly greater in surrounding soil than in roots (Table 2). The OTU richness was significantly different between sites but not between hosts and was higher in UTCBF (Table 2 and Table S7). Also, Shannon index was significantly different between sites, but not between species or compartments (Table 2 and Table S7). In total, 383 core AMF OTUs were detected, and the two host species shared 199 intraradical AMF OTUs (exclusively) across all sites (Online Resource 2).

**Table 2:** Alpha diversity of the root and soil arbuscular mycorrhizal fungal (AMF) communities of *Cryptomeria japonica* (Cj) and *Chamaecyparis obtusa* (Co)

Sites	Chiba (UTCBF)					Chichibu (UTCF)					Tanashi (UTTF)	
Host species	Cj		Co		Cj		Co		Cj		Co	
Compartments	Root	Soil	Root	Soil	Root	Soil	Root	Soil	Root	Soil	Root	Soil
Observed OTU richness^a)^	176 ± 18	200 ± 27	157 ± 17	191 ± 25	163 ± 18	228 ± 12	162 ± 34	211 ± 28	135 ± 21	197 ± 15	144 ± 21	198 ± 16
Observed Shannon index^a)^	3.02 ± 0.32	3.00 ± 0.38	2.87 ± 0.12	2.98 ± 0.15	3.02 ± 0.15	3.14 ± 0.24	2.95 ± 0.40	2.98 ± 0.31	2.61 ± 0.29	2.61 ± 0.18	2.79 ± 0.19	2.50 ± 0.21

^a)^Mean ± SE. Two-way ANOVA did not show a significant interaction effect between site and host species on any variable (Table S7). The OTU richness was significantly different between sites and between compartments. The Shannon index was different only between sites

There were 29 dominant OTUs (Table 3), which corresponded to six genera (*Acaulospora*, *Dominika*, *Glomus*, *Microkamienskia*, *Rhizophagus*, and *Sclerocystis*), two unknown clades, and some unknown Glomeraceae (Online Resource 3, Table 3). The CLAM detected 8 and 267 AMF OTUs significantly associated with a host (4 for Cj and 4 for Co) and a compartment (90 for root and 177 for soil), respectively (Online Resource 4, Table S8).

**Table 3:** Dominant operational taxonomic units (OTUs) in the arbuscular mycorrhizal fungi communities associated with *Cryptomeria japonica* (Cj) and *Chamaecyparis obtusa* (Co)

Dominant OTUs^a)^		Taxonomic affiliation				Relative abundance in each group of samples^f)^												CLAM^g)^	
Accession No.	Total abundance^b)^	NCBI or Maarj*AM*^c)^			Phylogenetic placement (Genus level)^e)^	Chiba (UTCBF)				Chichibu (UTCF)				Tanashi (UTTF)				Compartment association	Host association
						Cj		Co		Cj		Co		Cj		Co			
		No.	Genus^d)^	Per. Id		Root	Soil	Root	Soil	Root	Soil	Root	Soil	Root	Soil	Root	Soil		
MZ479763	2596	AB220170	*Acaulospora*	96.02	*Acaulospora*	-	-	-	-	0.022	-	-	-	0.031	-	0.021	-	Root	-
MZ479753	19922	VTX00166	*Glomus*	99.81	*Dominikia*	0.143	0.042	0.075	0.021	0.157	0.08	0.105	0.067	0.261	0.034	0.055	-	Root	Cj
MZ479784	825	VTX00186	*Glomus*	97.69	*Dominikia*	-	-	-	-	-	-	-	-	0.021	-	-	-	Root	Cj
MZ479756	8064	VTX00191	*Glomus*	100	*Glomus*	0.029	0.049	0.039	0.037	0.085	0.039	0.099	0.043	-	-	-	-	-	-
MZ479755	8963	VTX00122	*Glomus*	100	*Microkamienskia*	-	-	0.03	-	0.051	-	0.025	-	0.141	0.041	0.065	0.012	Root	-
MZ479752	26658	VTX00080	*Glomus*	99.62	*Rhizophagus*	0.245	0.07	0.29	0.105	0.12	0.044	0.187	0.055	0.095	0.021	0.192	0.032	Root	Cj & Co
MZ479757	6843	VTX00088	*Glomus*	99.42	*Rhizophagus*	0.031	-	0.086	-	0.101	0.016	0.059	-	0.024	-	0.027	-	Root	-
MZ479758	5511	VTX00084	*Glomus*	98.84	*Rhizophagus*	0.07	-	0.089	0.033	-	-	0.026	-	-	-	-	-	Root	-
MZ479760	4299	VTX00224	*Glomus*	99.81	*Rhizophagus*	0.039	0.021	0.04	-	0.023	-	0.044	0.029	-	-	-	-	Root & Soil	-
MZ479762	3039	VTX00084	*Glomus*	97.50	*Rhizophagus*	-	-	0.017	-	0.019	-	0.02	-	-	-	0.043	-	Root	-
MZ479765	2107	VTX00115	*Glomus*	100	*Rhizophagus*	0.017	-	-	-	0.032	-	0.037	-	-	-	-	-	Root	-
MZ479772	1696	VTX00291	*Glomus*	98.66	*Rhizophagus*	0.017	-	-	-	-	-	-	-	-	-	-	-	Root & Soil	-
MZ479776	1407	VTX00126	*Glomus*	98.46	*Rhizophagus*	-	0.019	-	-	-	0.016	-	0.03	-	-	-	-	Soil	-
MZ479775	1509	VTX00223	*Glomus*	97.89	*Sclerocystis*	0.017	-	0.025	-	-	-	-	-	-	-	-	-	-	-
MZ479770	1814	AJ871272	unclassified	85.71	Unknown clade 1	-	0.06	-	0.023	-	-	-	-	-	-	-	-	Soil	-
MZ479777	1352	AJ506090	unclassified	96.94	Unknown clade 1	-	0.043	-	-	-	-	-	-	-	-	-	-	-	-
MZ479786	757	AJ506090	unclassified	96.94	Unknown clade 1	-	-	-	0.027	-	-	-	-	-	-	-	-	Soil	-
MZ479751	44575	VTX00444	*Paraglomus*	99.04	Unknown clade 2	-	0.272	-	0.258	-	0.336	-	0.347	0.029	0.47	-	0.505	Soil	-
MZ479761	3219	VTX00444	*Paraglomus*	96.14	Unknown clade 2	-	0.027	-	0.064	-	0.018	-	0.014	-	0.022	-	0.02	Soil	-
MZ479767	2044	VTX00444	*Paraglomus*	96.56	Unknown clade 2	-	-	-	-	-	0.021	-	0.02	-	0.02	-	0.019	Soil	-
MZ479773	1640	VTX00444	*Paraglomus*	96.55	Unknown clade 2	-	-	-	-	-	-	-	-	-	0.041	-	0.013	Soil	-
MZ479774	1547	VTX00444	*Paraglomus*	97.12	Unknown clade 2	-	-	-	-	-	0.015	-	0.014	-	0.014	-	0.015	Soil	-
MZ479780	978	VTX00444	*Paraglomus*	95.96	Unknown clade 2	-	-	-	-	-	-	-	-	-	0.016	-	-	Soil	-
MZ479785	822	VTX00444	*Paraglomus*	96.15	Unknown clade 2	-	-	-	0.019	-	-	-	-	-	-	-	-	Soil	-
MZ479759	4544	VTX00124	*Glomus*	99.04	Unknown Glomeraceae	-	-	-	-	-	-	-	-	0.022	-	0.139	0.015	Root	Co
MZ479771	1783	AF480154	uncultured	95.63	Unknown Glomeraceae	-	-	-	-	-	-	-	-	-	-	0.052	0.011	Root	Co
MZ479782	895	VTX00124	*Glomus*	96.77	Unknown Glomeraceae	-	-	-	-	-	-	-	-	-	-	0.029	-	Root	Co
MZ479754	19116	VTX00219	*Glomus*	99.42	Unknown Glomeraceae	0.107	0.103	0.054	0.078	0.104	0.089	0.075	0.078	0.059	0.061	0.118	0.108	-	-
MZ479768	1893	VTX00214	*Glomus*	100	Unknown Glomeraceae	-	-	-	-	-	-	-	-	0.059	-	-	-	-	-

^a)^The top 10 most abundant AMF OTUs per group of samples. ^b)^Total abundance after rarefaction. ^c)^Accessions of the closest matches obtained from the NCBI or MaarjAM database: taxa were assigned to OTUs only when from the database, both query cover and percent of identity with the match were higher or equal to 95%; “Per. Id, the percentage of identity to the closest match. ^d)^Unclassified, OTUs for which the taxon assignment criteria were not met; Uncultured, OTUs for which the best matches were described as such in the database. ^e)^Results of maximum likelihood phylogenetic placement of OTUs (**Online Resource** **3**). ^f)^-, OTU not among the top 10 most abundant OTUs in the corresponding group of samples. ^g)^Classification based on the multinomial species classification method (CLAM). -, the OTU was not successfully classified

The extraradical but not the intraradical AMF community showed a significantly association with environmental variables altogether (Table S9). Individually, only soil pH was significantly correlated with the root and soil AMF communities, and the correlation with the soil community was stronger than that with the root community. In addition, the correlation of root community with that of soil community was not significant.

The genus-level composition and structure of the AMF community were significantly different between sites and compartments but not between hosts, and there was a significant interaction between site and compartment (Table S10). Based on a BLAST search and phylogenetic analysis, we detected 15 AMF genera in the community, predominantly *Glomus* and *Paraglomus* (Table 4). *Glomus* and *Acaulospora* were significantly associated with the root, while *Paraglomus* and *Redeckera* were in the surrounding soil (Table 5).

**Table 4:** Taxonomic composition and structure of the arbuscular mycorrhizal fungi (AMF) communities associated with *Cryptomeria japonica* (Cj) and *Chamaecyparis obtusa* (Co) showing relative abundance of AMF taxa in each group of samples

Order and Family^a)^	Genus^a), b)^	Relative abundance^c)^											
		Chiba (UTCBF)				Chichibu (UTCF)				Tanashi (UTTF)			
		Cj		Co		Cj		Co		Cj		Co	
		Root	Soil	Root	Soil	Root	Soil	Root	Soil	Root	Soil	Root	Soil
Archaeosporales													
Archaeosporaceae	*Archaeospora*	0.000	0.000	-	0.000	0.010	0.017	0.010	0.008	0.008	0.013	0.009	0.004
Diversisporales													
Acaulosporaceae	*Acaulospora*	0.002	0.002	0.003	0.002	0.024	0.006	0.021	0.010	0.035	0.017	0.023	0.003
Diversisporaceae	*Diversispora*	0.015	0.006	0.000	0.004	0.002	0.001	0.002	0.001	0.001	0.001	0.000	0.000
Gigasporaceae	*Gigaspora*	-	0.000	0.000	0.000	0.000	-	-	-	0.000	0.000	0.000	0.000
	*Scutellospora*	0.001	0.001	0.001	0.001	0.002	0.001	0.002	0.004	0.001	0.004	0.000	0.000
Glomerales													
Claroideoglomeraceae	*Claroideoglomus*	-	0.000	0.000	0.000	0.001	0.002	0.001	0.000	0.000	0.000	-	0.000
Glomeraceae	*Funneliformis*	0.000	-	-	-	-	0.000	-	-	0.000	-	0.000	0.000
	*Glomus*	0.912	0.422	0.925	0.415	0.889	0.412	0.897	0.425	0.864	0.239	0.848	0.254
	*Redeckera*	-	-	-	0.000	0.000	-	-	0.000	-	0.000	-	0.007
	*Rhizophagus*	0.000	-	0.000	0.000	0.001	0.000	0.001	0.000	0.000	0.001	0.000	0.000
	*Septoglomus*	-	-	-	-	0.000	0.000	-	-	-	-	-	0.000
Paraglomerales													
Paraglomeraceae	*Paraglomus*	0.031	0.424	0.019	0.451	0.030	0.509	0.031	0.509	0.044	0.697	0.040	0.689
Unknown	Unclassified	0.012	0.120	0.016	0.095	0.009	0.031	0.007	0.023	0.008	0.012	0.006	0.014
	Uncultured	0.026	0.025	0.035	0.030	0.032	0.020	0.029	0.020	0.039	0.015	0.073	0.029

^a)^Taxonomic information updated according to the list of AMF species available at http://amf-phylogeny.com. ^b)^Unclassified, OTUs for which the taxon assignment criteria were not met in the NCBI and MaarjAM databases; uncultured, OTUs for which the best matches were described as such in the databases. The phylogenetic analysis detected three genera not shown in this table (Table 3).^c)^ Community composition obtained by blasting the representative amplicon sequences of the OTUs against the NCBI and MaarjAM databases. -, taxa not detected in the corresponding group of samples.

**Table 5:** Associations of arbuscular mycorrhizal fungi (AMF) genera with host (*Cryptomeria japonica* and *Chamaecyparis obtusa*) and compartment (root and soil) based on multinomial species classification method (CLAM)

A: CLAM for classification of AMF genera into compartments of the rhizosphere				
AMF genus	Total abundance	Abundance in root	Abundance in soil	Class
*Glomus*	139883	100425	39458	Root
*Acaulospora*	2995	2184	811	Root
*Archaeospora*	1550	719	831	Root & Soil
*Diversispora*	533	289	244	Root & Soil
*Scutellospora*	333	131	202	Root & Soil
*Rhizophagus*	79	42	37	Root & Soil
*Claroideoglomus*	73	28	45	Root & Soil
*Gigaspora*	39	22	17	Root & Soil
*Funneliformis*	15	13	2	Root & Soil
*Paraglomus*	67627	3759	63868	Soil
*Redeckera*	164	2	162	Soil
*Septoglomus*	10	1	9	Not classified
B: CLAM for classification of AMF genera into host species				
AMF genus	Total abundance	Total abundance in Cj	Total abundance in Co	Class
*Diversispora*	289	246	43	Cj
*Glomus*	100425	48973	51452	Cj and Co
*Paraglomus*	3759	2018	1741	Cj and Co
*Acaulospora*	2184	1320	864	Cj and Co
*Archaeospora*	719	384	335	Cj and Co
*Scutellospora*	131	83	48	Cj and Co
*Rhizophagus*	42	20	22	Cj and Co
*Claroideoglomus*	28	15	13	Cj and Co
*Gigaspora*	22	6	16	Cj and Co
*Funneliformis*	13	7	6	Not classified
*Redeckera*	2	2	0	Not classified
*Septoglomus*	1	1	0	Not classified

### AMF Root Colonization

All analyzed root samples showed AMF colonization (MF = 100%). Arbuscles, hypha, and vesicles were observed in Cj and Co (Online Resource 5). Hyphae were most evident in stained roots (up to 75%), followed by vesicles and arbuscules, with the latter being very rare (< 13%). We found significant site-dependent variation in AC between species (Table S11). The HC, however, was significantly different between sites and hosts, without a significant interaction. The AC value was higher in UTCBF with Cj, and with HC in Cj (Table 6). Neither factor significantly affected the VC (Table S11). The HC correlated positively with soil OTU richness and soil Shannon index; the AC correlated positively with the root OTU richness and soil Shannon index (Table S12).

**Table 6:** Intensity of colonization of *Cryptomeria japonica* (Cj) and *Chamaecyparis obtusa* (Co) roots by morphology (arbuscules, hyphae, and vesicles)

AMF Mycorrhization measures^a)^	Chiba (UTCBF)		Chichibu (UTCF)		Tanashi (UTTF)	
	Cj	Co	Cj	Co	Cj	Co
Arbuscular colonization (AC)^b)^	12.64 ± 5.87 a	0.37 ± 0.74 b	10.21 ± 8.66 ab	7.08 ± 4.83 ab	1.09 ± 0.69 ab	2.59 ± 3.38 ab
Hyphal colonization (HC)^c)^	36.9 ± 15.99	5.74 ± 7.65	74.52 ± 15.56	19.13 ± 19.04	25.33 ± 16.45	7.05 ± 7.72
Vesicular colonization (VC)^d)^	11.67 ± 6.46	16.49 ± 6.69	12.7 ± 6.88	12.5 ± 5.12	11.98 ± 7.18	18.35 ± 7.05

^a)^*AMF*, arbuscular mycorrhizal fungi. Mean ± SE is shown for the mycorrhization intensity variables (AC, HC, and VC). ^b)^There was a significant interaction effect between forest and host; means followed by the same letter do not differ significantly by Tukey HSD test following two-way ANOVA. ^c)^No significant interaction between site and host species but each factor exerted a significant effect (Table S11). ^d)^Neither factor or their interaction showed a significant effect (Table S11)

## Discussion

Cupressaceaous conifers, which have AMF, have been poorly investigated for their mycorrhizal partners. Before this study, no quantitative assessment of AMF colonization of Co roots had been conducted, unlike Cj. Because the formation of arbuscules, hypha, and vesicles differs among AMF species [29], and these components play different roles in symbiosis [30], information on how each morphological type colonizes the roots of tree species is crucial to understanding the ecophysiology of AMF colonization. Our results indicated that whether planted separately or together, Cj and Co are differently colonized by AMF.

Soil conditions, mainly pH, play a crucial role in AMF symbiosis [31]. The soil pH and EC were significantly different between Cj and Co in this study. These results could explain the differences between Cj and Co in terms of AMF root colonization. The root AMF species richness had a significant correlation with AC and that of the soil with AC and HC, suggesting that the AMF inoculum in soil determines AMF root colonization (Table S12).

**Fig. 2:**
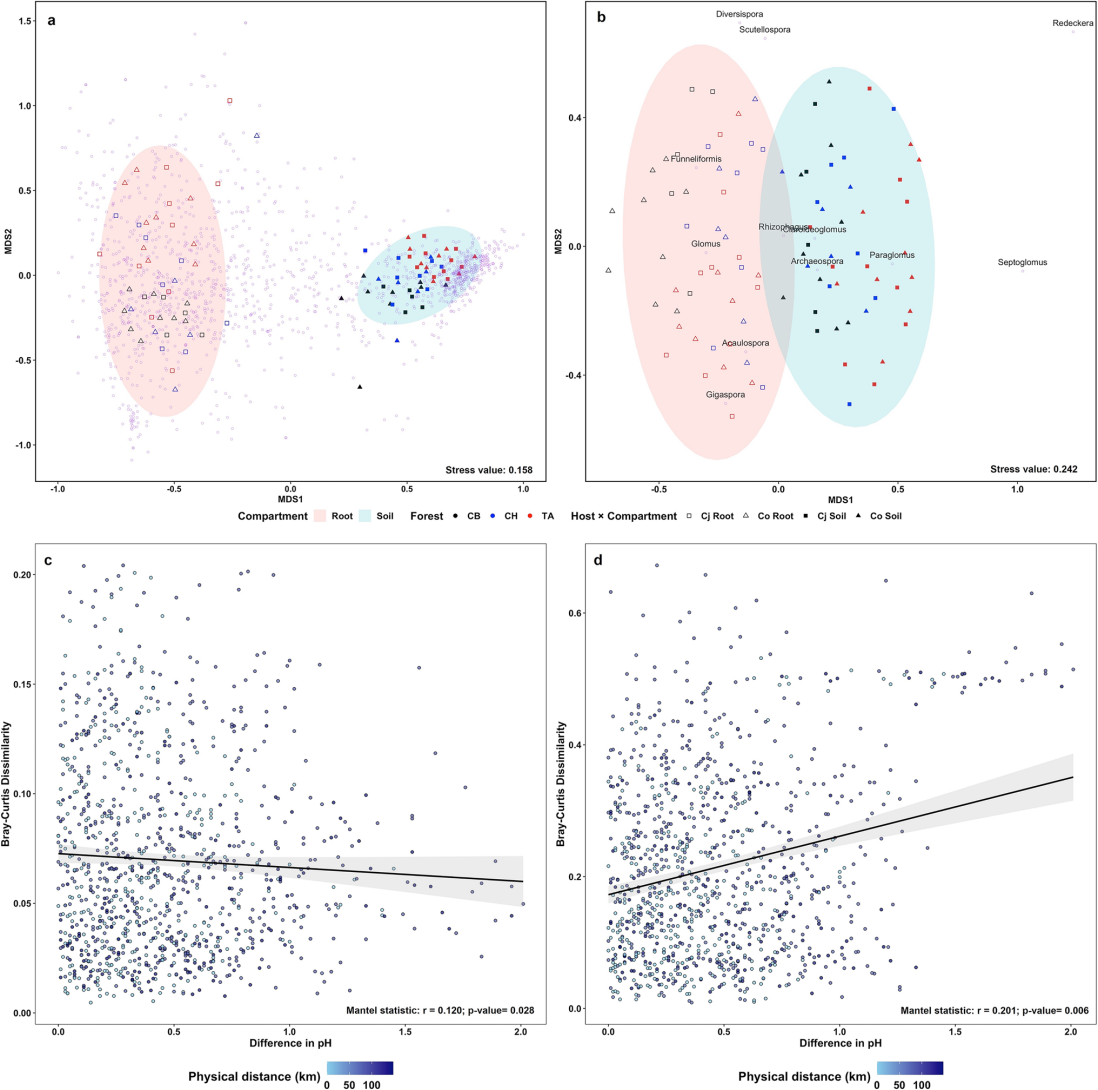
Multidimensional scaling plots of the intra- and extraradical communities of arbuscular mycorrhizal fungi (AMF) associated with *Cryptomeria japonica* (Cj) and *Chamaecyparis obtusa* (Co) collected from three sites in Japan. **a** and **b** Sample groupings by compartment (root and soil) at the OTU and genus levels, respectively. *Glomus* and *Acaulospora* were significantly associated with roots whereas soil was significantly associated with *Paraglomus* and *Redeckera* (Table 5). **c** and **d** The effects of soil pH and geographical separation on the root and soil AMF communities, respectively. Geographical separation significantly affected the soil, but not the root AMF community; pH correlated significantly with both communities but had a stronger effect on the soil than the root community (Table S9)

The composition and structure of the intraradical AMF communities of Cj and Co differed significantly from those in the surrounding soil (Fig. 2). These results are consistent with most previous findings [7, 9, 32, 33]. By contrast, the non-significant difference reported by Djotan et al. [10] between the root and surrounding soil AMF communities associated with Cj may be a result of the small sample size and/or sampling season. The AMF communities in roots and corresponding surrounding soil can be affected by methodological differences [8]. Here, root and surrounding soil samples were collected simultaneously under the same trees in different physical environments (Table S1). Also, obtaining DNA from root and soil samples overcomes the imperfect proxy problem raised by Stevens et al. [34]. Thus, the difference between the root and soil AMF communities could be attributed to a strategic root-soil exploration and biomass allocation in AMF [14], as well as the selection of AMF inocula in soil by their hosts [12]. AMF colonizing roots appear to be protected from environmental stresses present in soil. This assumption is supported by the Mantel test results which showed that soil pH and geographical separation have stronger effects on soil than the root AMF community (Fig. 2, Table S9). Selection and protection by the host explain the more homogenous AMF community in the root than soil across sites (Fig. 2), and why AMF communities reflect local environmental conditions and spatial distance between sites [35]. Our result is consistent with the report of Stevens et al. [34] that root and soil AMF communities respond differently to environmental factors. In addition, the variation in soil AMF community does not necessarily induce variation in the related root AMF community (Table S9). Therefore, the host plants act as biotic (selection and physiological influence) and abiotic (physical protection against direct effects of environmental factors) filters and alter the AMF community composition between the soil and the root.

We detected three classes of AMF OTUs or genera using CLAM, the root explorers (more abundant in roots than in soil), the soil explorers (more abundant in soil than in roots), and the explorers of both, thereby validating the hypothesis of strategic taxon-based colonization in the AMF community (Online Resource 4, Table S8). The root versus soil fungal exploration patterns, which suggest a topological connection between root and soil, may sustain the mutual benefits to the host and symbionts. *Glomus* and *Acaulospora* were significantly associated with the roots, while *Paraglomus* and *Redeckera* were significantly associated with the soil (Fig. 2, Table 5). These results are consistent with a report that different AMF taxa are differently distributed in the root and soil during their life history [36]. Glomeraceae and *Glomus* first infest and colonize roots, where they rapidly become the most abundant AMF symbionts [7, 14], whereas Paraglomeraceae and *Paraglomus* are reportedly more abundant in soil [7, 37].

In this study, there were more AMF OTUs in the surrounding soil than in the roots (Online Resource 1). However, other studies reported different AMF OTU richness values and community similarities between roots and surrounding soil [7]. This discrepancy can be explained by the use of different hosts, sites, seasons, AMF quantification proxies, and overall approaches [2, 4, 8], which varied among prior studies but were controlled in this work. In previous studies of the AMF communities of Cj and Co [19, 20, 38], root and soil OTU richness were not both evaluated, thus precluding comparison of intra- and extraradical AMF communities. In Venn diagrams, the number of AMF OTUs exclusive to the roots of Cj or Co decreased when data from all sites were considered (Online Resource 2). This indicates spatial OTU turnover in the intraradical AMF community of Cj and Co and supports the spatiotemporal hypothesis of AMF community dynamics [39, 40]. The lower Shannon index values in UTTF than UTCBF and UTCF (Table 2) support the unification of island biogeography and niche theories [41].

The AMF community was significantly different between sites and hosts (Table S4). The significant differences in AMF communities among sites could be explained by differences in site-related factors and variables (Table 1 and Table S1). Similar variations were reported for secondary forests and Co plantations in Japan [38]. They found that the plant community composition affected the AMF community composition, which also varied between sites. We also detected differences in the understory plant communities among sites, which supports their conclusion. In contrast, Matsuda et al. [20] found no variation among sites in the AMF communities in Cj roots. The size of the amplicon used by Matsuda et al. [20] to characterize the AMF community was smaller than in this study, which probably failed to capture the variation in molecular diversity of the AMF community associated with Cj between their study sites. The host effect was significant only in UTTF, where Cj and Co plantations were adjacent and physically separated (Table 1, and Tables S1 and S6). These results suggest that Cj and Co may be involved in a mycorrhizal network in which they share AMF symbionts when in proximity (Tables S6 and S9). These findings support host-related variation in AMF communities [12] and the greater effect of space than host identity [40] on AMF communities.

Among the 15 AMF genera detected in this study using the GenBank and Maarj*AM* databases (Table 4) and phylogenetic analysis (Online Resource 3), *Glomus* and *Paraglomus* were the most abundant in the AMF community (Table 3). Glomus or Glomeraceae was most abundant in the majority of previous investigations of AMF communities associated with Cj or Co [10, 20, 38] or in many other host plants in different regions globally [35, 42]. In this study, we further provided a compared composition and structure of AMF community between root and surrounding soil. MZ479751 (VTX00444), MZ479752 (VTX00080), MZ479753 (VTX00166), and MZ479754 (VTX00219) were the four most dominant OTUs recorded in the current study (Table 3). According to the global distribution of virtual AMF taxa based on Maarj*AM* database, all but MZ479751 were globally distributed, suggesting that the corresponding AMF species are cosmopolitan. In contrast, MZ479751 which was the most dominant OTUs recorded in our study was previously recorded in Estonia only. Therefore, the corresponding AMF species may have a restrain geographical distribution or data on the species are not submitted to publicly available databases. In this study, several dominant OTUs corresponded to the same virtual taxa defined in the Maarj*AM* database (Table 4). Miyake et al. [38] used the same OTU clustering threshold (97%) and reported similar results. Compared to previous studies of Cj and Co AMF communities, our work yielded larger numbers of AMF OTUs and dominant AMF OTUs, possibly because of the sampling design. In addition, Japan has ecosystems with large numbers of AMF taxa. For example, Öpik et al. [43] indicated in a review that Saito et al. [44] recorded the second-greatest AMF taxon richness (24 AMF taxa) from two temperate grassland sites in Japan. We recorded 15 taxa from three sites in planted Cj and Co forests. So, contrary to the conclusion of Miyake et al. [38], AMF communities in Japan are not composed of small numbers of taxa.

## Conclusion

In this study, we validated the hypothesis of strategic exploration of the rhizosphere by AMF and described the associations in the AMF community of roots and the surrounding soil. Root and soil AMF communities responded differently to environmental factors, suggesting that soil AMF taxa directly reflect the physical condition of the soil, whereas root AMF taxa are selected and protected by the host. This strategic root versus soil association pattern in the AMF community may sustain the mutual benefits to host and symbionts. Also, host plants may collaborate and share an AMF community via proximal networks, but this disappears upon geographical separation.

## Supplementary Information

ESM 1 **Online Resource 1** Accumulation curves of AMF OTUs detected in *Cryptomeria japonica* (Cj) and *Chamaecyparis obtusa* (Co), collected from three sites in Japan. Normalized community data was used to build these curves, 2411 Glomeromycotan amplicon sequences per sample. Despite the differences in the number of samples per group, it is noticeable that OTU richness of the arbuscular mycorrhizal fungi (AMF) community was higher in soil than roots

ESM 2 **Online Resource 2** Venn diagrams of shared operational taxonomic units (OTUs) in roots and soil communities of arbuscular mycorrhizal fungi (AMF) associated with *Cryptomeria japonica* (Cj) and *Chamaecyparis obtusa* (Co), collected from three sites in Japan. Notice that the number of OTUs exclusively in roots of Cj (Cj Root) or Co (Co Root) has reduced considerably when data from all sites were considered

ESM 3 **Online Resource 3** Phylogenetic tree for the placement of the dominant (top 10 most abundant) operational taxonomic units (OTUs) in the intra- and extraradical communities of arbuscular mycorrhizal fungi (AMF) associated with *Cryptomeria japonica* (Cj) and *Chamaecyparis obtusa* (Co) collected from three sites in Japan. Maximum likelihood tree was built using the representative sequences of the dominant OTUs (29 nucleotide sequences) and 53 reference nucleotide sequences downloaded from NCBI GenBank and Maarj*AM* databases. Best model and parameters were selected with automatic model finder in IQ-TREE 2. SH-aLRT test and ultrafast bootstrap (UFBoot) over 1000 randomizations were performed and UFboot ≥ 95% are shown at the nodes where SH-aLRT ≥ 80%. Accessions of the dominant OTUs (in bold) and scientific names of reference sequences followed by their accessions were used for labeling. All sequences contained an average of 550 bp of the small subunit ribosomal DNA between the primer pairs NS31 and AM1 (EPS 1595 kb)

ESM 4 **Online Resource 4** Classification of AMF OTUs in two habitats using multinomial species classification method (CLAM) for the host (*Cryptomeria japonica* and *Chamaecyparis obtusa*) and the compartment (root and soil). Only root samples were used for the host-related classification while root and soil samples were used for the compartment-related classification. Generalist, similarly abundant in both habitats; x specialist, more abundant in the habitat x than the other; Too rare, the OTUs is too rare to be classified with confidence

ESM 5 **Online Resource 5** Anatomical structures of arbuscular mycorrhizal fungi (AMF) in stained roots of *Cryptomeria japonica* (Cj, a-c) and *Chamaecyparis obtusa* (Co, d-f). a and d show vesicles and hyphae, respectively while others show different morphologies of arbuscules

ESM 6 Table S1 Summary of the study sites a) UTCBF, Chiba; UTCF, Chichibu; UTTF, Tanashi; MAP, mean annual precipitation; MAT, mean annual temperature; Cj, *Cryptomeria japonica*; Co, *Chamaecyparis obtusa*. b) The UTCBF and UTCF are mixed plantations of Cj and Co. The UTTF site is an adjacent Cj plantation and Co plantation. Table S2 Understory plant community composition of the study sites. a) + refers to the presence at the corresponding site. Table S3 Analyses of variance on soil pH, soil electrical conductivity, and host diameter at breast height a) Variables are soil pH, soil electrical conductivity (EC), and diameter at breast height (DBH) of the host tree. Factors are Site and Host. Sites are Chiba (UTCBF), Chichibu (UTCF), and Tanashi (UTTF). Hosts are *Cryptomeria japonica* (Cj) and *Chamaecyparis obtusa* (Co). Table S4 Permanova on the arbuscular mycorrhizal fungi (AMF) community at the OTU level. a) Sites are Chiba (UTCBF), Chichibu (UTCF), and Tanashi (UTTF). Hosts are *Cryptomeria japonica* (Cj) and *Chamaecyparis obtusa* (Co). Compartments are root and surrounding soil. Table S5 Mantel test showing the biotic and abiotic effects on the root and soil AMF communities associated with *Cryptomeria japonica* (Cj) and *Chamaecyparis obtusa* (Co). Significant effects (p-value < 0.05) are in bold. a) Sites are Chiba (UTCBF), Chichibu (UTCF), and Tanashi (UTTF). Factors are Host and Compartment. Hosts are *Cryptomeria japonica* (Cj) and *Chamaecyparis obtusa* (Co). Compartments are root and surrounding soil. Table S6 Analysis of root and soil AMF communities similarities between *Cryptomeria japonica* (Cj) and *Chamaecyparis obtusa* (Co). ANOSIM p-value < 0.05 (in bold) refers to significantly different communities. Table S7 Analyses of variance on the alpha diversity indices of arbuscular mycorrhizal fungi (AMF) community. a) Variables are number of operational taxonomic units (NOTUs) and Shannon index. Factors are Site, Host, and Compartment. Sites are Chiba (UTCBF), Chichibu (UTCF), and Tanashi (UTTF). Hosts are *Cryptomeria japonica* (Cj) and *Chamaecyparis obtusa* (Co). Compartments are root and surrounding soil. Table S8 Association of arbuscular mycorrhizal fungi (AMF) with host species (*Cryptomeria japonica*, Cj; and *Chamaecyparis obtusa*, Co) or compartments of the rhizosphere (Root and Soil) based on the multinomial species classification method (CLAM). Table S9 Mantel test showing the biotic and abiotic effects on the root and soil AMF communities associated with *Cryptomeria japonica* (Cj) and *Chamaecyparis obtusa* (Co). Significant effects (p-value < 0.05) are in bold. Table S10 Permanova on the arbuscular mycorrhizal fungi (AMF) community at the genus level. Table S11 Analyses of variance on root colonization by type of arbuscular mycorrhizal fungi (AMF) morphotypes. Table S12 Pearson correlations showing the association of root and soil conditions with the root colonization of *Cryptomeria japonica* (Cj) and *Chamaecyparis obtusa* (Co). Correlation values are followed by the significance probability in parentheses. Significant correlations (p-value < 0.05) are in bold
