# Correction to: Paired root-soil samples and metabarcoding reveal taxon-based colonization strategies in arbuscular mycorrhizal fungi communities in Japanese cedar and cypress stands

**DOI:** 10.1007/s00248-023-02246-2

**Published:** 2023-05-24

**Authors:** Akotchiffor Kevin Geoffroy Djotan, Norihisa Matsushita, Kenji Fukuda

**Affiliations:** Graduate School of Agricultural and Life Sciences (Laboratory of Forest Botany), University of Tokyo, 1‑1‑1, Yayoi, Bunkyo‑ku, Tokyo 113‑8657 Japan

**Correction to: Microbial Ecology, 1–14, 2023**

**https://doi.org/10.1007/s00248-023-02223-9**

There are lines and text misalignment in Table 2:

**Table 2:** Alpha diversity of the root and soil arbuscular mycorrhizal fungal (AMF) communities of *Cryptomeria japonica* (Cj) and *Chamaecyparis obtusa* (Co)

Sites	Chiba (UTCBF)				Chichibu (UTCF)				Tanashi (UTTF)			
Host species	Cj		Co		Cj		Co		Cj		Co	
Compartments	Root	Soil	Root	Soil	Root	Soil	Root	Soil	Root	Soil	Root	Soil
Observed OTU richness a)	176 ± 18	200 ± 27	157 ± 17	191 ± 25	163 ± 18	228 ± 12	162 ± 34	211 ± 28	135 ± 21	197 ± 15	144 ± 21	198 ± 16
Observed Shannon index a)	3.02 ± 0.32	3.00 ± 0.38	2.87 ± 0.12	2.98 ± 0.15	3.02 ± 0.15	3.14 ± 0.24	2.95 ± 0.40	2.98 ± 0.31	2.61 ± 0.29	2.61 ± 0.18	2.79 ± 0.19	2.50 ± 0.21

^a)^ Mean ± SE. Two-way ANOVA did not show a significant interaction effect between site and host species on any variable (Table S7). The OTU richness was significantly different between sites and between compartments. The Shannon index was different only between sites

In the footnote of Tables 3 and 4, « MaarjAM » needs to be corrected to « Maarj*AM* ».

In Table 5, *Septoglomus* is misaligned with the other genera. Also, dashed lines should separate data for the compartment and host classifications.

**Table 5:** Associations of arbuscular mycorrhizal fungi (AMF) genera with host (*Cryptomeria japonica* and *Chamaecyparis obtusa*) and compartment (root and soil) based on multinomial species classification method (CLAM)

**A: CLAM for classification of AMF genera into compartments of the rhizosphere**				
AMF genus	Total Abundance	Abundance in Root	Abundance in Soil	Class
*Glomus*	139,883	100,425	39,458	Root
*Acaulospora*	2995	2184	811	Root
*Archaeospora*	1550	719	831	Root & Soil
*Diversispora*	533	289	244	Root & Soil
*Scutellospora*	333	131	202	Root & Soil
*Rhizophagus*	79	42	37	Root & Soil
*Claroideoglomus*	73	28	45	Root & Soil
*Gigaspora*	39	22	17	Root & Soil
*Funneliformis*	15	13	2	Root & Soil
*Paraglomus*	67,627	3759	63,868	Soil
*Redeckera*	164	2	162	Soil
*Septoglomus*	10	1	9	Not classified
**B: CLAM for classification of AMF genera into host species**				
AMF genus	Total Abundance	Total Abundance in Cj	Total Abundance in Co	Class
*Diversispora*	289	246	43	Cj
*Glomus*	100,425	48,973	51,452	Cj and Co
*Paraglomus*	3759	2018	1741	Cj and Co
*Acaulospora*	2184	1320	864	Cj and Co
*Archaeospora*	719	384	335	Cj and Co
*Scutellospora*	131	83	48	Cj and Co
*Rhizophagus*	42	20	22	Cj and Co
*Claroideoglomus*	28	15	13	Cj and Co
*Gigaspora*	22	6	16	Cj and Co
*Funneliformis*	13	7	6	Not classified
*Redeckera*	2	2	0	Not classified
*Septoglomus*	1	1	0	Not classified

Tables S10 and S11 are missing the footnotes as per the details in ESM 6.

Footnote of Table S10

^a)^ Sites are Chiba (UTCBF), Chichibu (UTCF), and Tanashi (UTTF). Hosts are *Cryptomeria japonica* (Cj) and *Chamaecyparis obtusa* (Co). Compartments are root and surrounding soil.

Footnote of Table S11

^a)^ Variables are Arbuscular (AC), Hyphal (HC), and Vesicular Colonization (VC) by Arbuscular Mycorrhizal Fungi (AMF). Factors are site and host species. Sites are Chiba (UTCBF), Chichibu (UTCF), and Tanashi (UTTF). Hosts are *Cryptomeria japonica* (Cj) and *Chamaecyparis obtusa* (Co).

## Data Availability

The datasets generated during and/or analysed during the current study are available from the corresponding author on reasonable request.

