# Supplementary figures and images for: Paired Root-Soil Samples and Metabarcoding Reveal Taxon-Based Colonization Strategies in Arbuscular Mycorrhizal Fungi Communities in Japanese Cedar and Cypress Stands
Source: Microb Ecol. 2023 Apr 28;86(3):2133–46. doi: 10.1007/s00248-023-02223-9 (PMC10497666; doi:10.1007/s00248-023-02223-9)

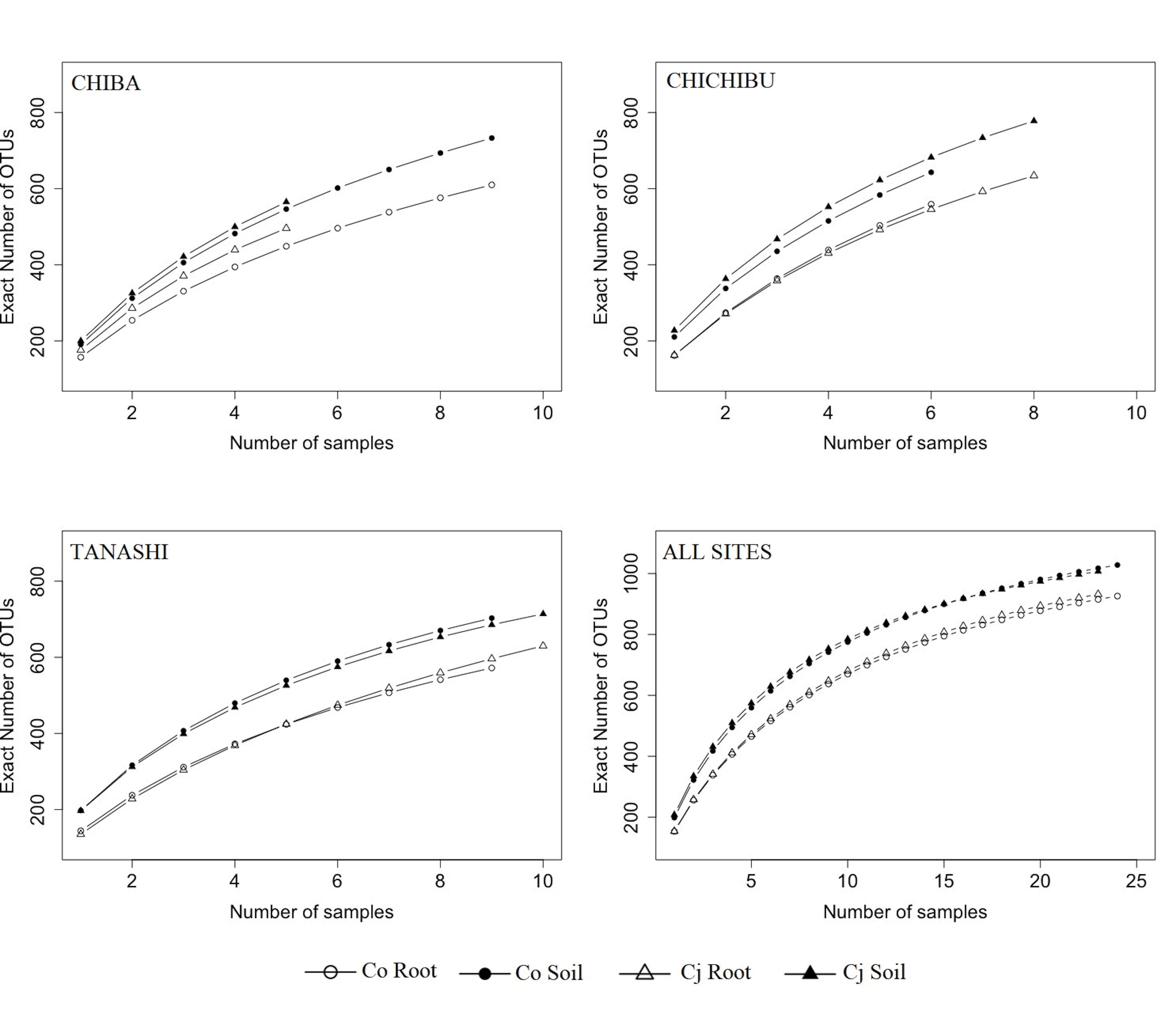

Supplement: Supplementary file 1 — Online Resource 1 Accumulation curves of AMF OTUs detected in Cryptomeria japonica (Cj) and Chamaecyparis obtusa (Co), collected from three sites in Japan. Normalized community data was used to build these curves, 2411 Glomeromycotan amplicon sequences per sample. Despite the differences in the number of samples per group, it is noticeable that OTU richness of the arbuscular mycorrhizal fungi (AMF) community was higher in soil than roots [file 248_2023_2223_MOESM1_ESM.jpeg]

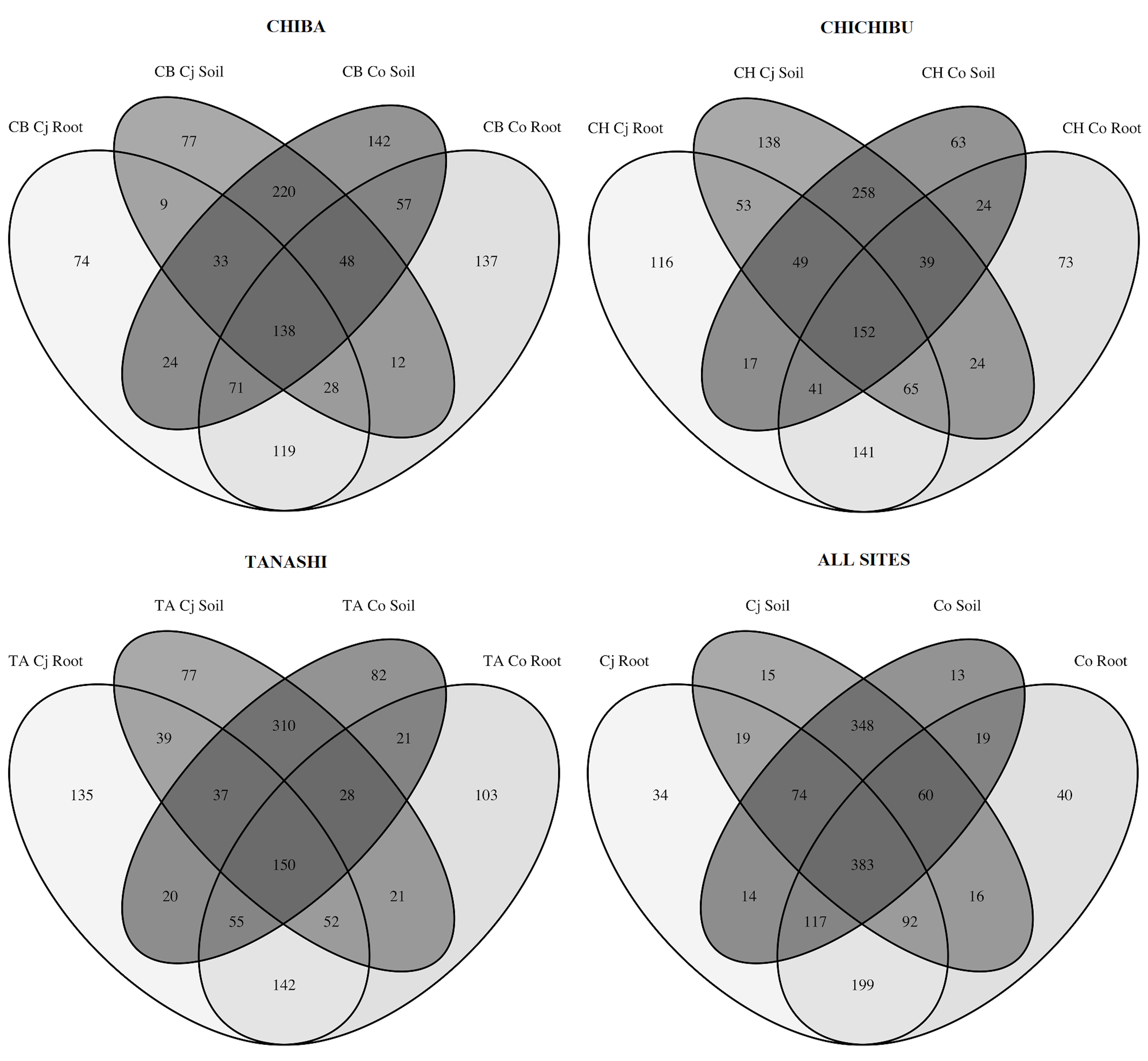

Supplement: Supplementary file 2 — Online Resource 2 Venn diagrams of shared operational taxonomic units (OTUs) in roots and soil communities of arbuscular mycorrhizal fungi (AMF) associated with Cryptomeria japonica (Cj) and Chamaecyparis obtusa (Co), collected from three sites in Japan. Notice that the number of OTUs exclusively in roots of Cj (Cj Root) or Co (Co Root) has reduced considerably when data from all sites were considered [file 248_2023_2223_MOESM2_ESM.jpeg]

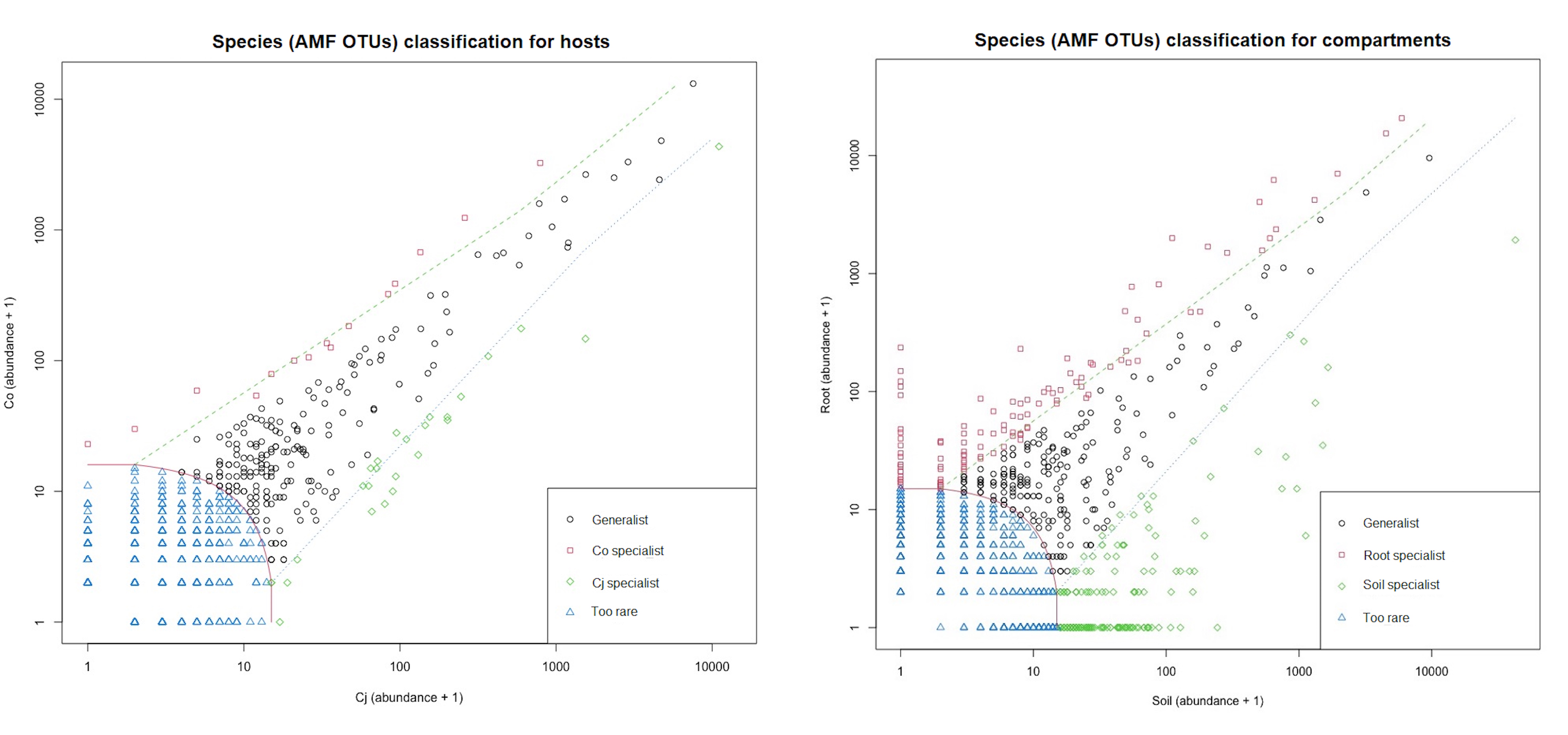

Supplement: Supplementary file 4 — Online Resource 4 Classification of AMF OTUs in two habitats using multinomial species classification method (CLAM) for the host (Cryptomeria japonica and Chamaecyparis obtusa) and the compartment (root and soil). Only root samples were used for the host-related classification while root and soil samples were used for the compartment-related classification. Generalist, similarly abundant in both habitats; x specialist, more abundant in the habitat x than the other; Too rare, the OTUs is too rare to be classified with confidence [file 248_2023_2223_MOESM4_ESM.jpg]
